# It Takes a Village: Patient Satisfaction in a Specialized Atypical Parkinsonism Interdisciplinary Clinic

**DOI:** 10.1177/23982128261456452

**Published:** 2026-05-27

**Authors:** Margaret Ivancic, Jessica Shurer, Hailey Daly, Nina Browner, Miriam Sklerov

**Affiliations:** 1 Department of Neurology, University of North Carolina School of Medicine, North Carolina, USA; 2 CurePSP, Inc, New York, USA

**Keywords:** movement disorders, parkinsonism, atypical parkinsonism, interdisciplinary clinic, progressive supranuclear palsy, multiple system atrophy, corticobasal syndrome

## Abstract

**Background & Objectives:**

Atypical parkinsonism syndromes (multiple system atrophy (MSA), progressive supranuclear palsy (PSP), and corticobasal syndrome (CBS)) are life-limiting neurodegenerative disorders with complex motor and nonmotor symptoms. A person-centered, coordinated, multidisciplinary approach may provide optimal care. Interdisciplinary care models in complex neurologic diseases are shown to improve disease outcomes. It is unknown whether specialized interdisciplinary care benefits people with atypical parkinsonism. Our study sought to determine patient satisfaction with a specialized atypical parkinsonism interdisciplinary clinic.

**Methods:**

A retrospective, cross-sectional analysis was conducted of anonymous satisfaction surveys from patients who attended the UNC atypical parkinsonism interdisciplinary clinic from 10/2017-3/2024. The survey was divided into four subcategories – Services, Results, Clinicians, and Overall Experience. Responses were converted to numerical values for statistical analysis. Linear regression was used to determine predictors of overall experience scores. Qualitative data was analyzed and categorized into themes.

**Results:**

51.4% of all clinic attendees were diagnosed with PSP, 36.5% with MSA, and 9.5% with CBS. 39 of 74 (52.7%) satisfaction surveys were completed. Survey responses indicated high levels of satisfaction across all categories. Responses in the services category were the strongest predictor of variability in overall experience scores. Common themes from qualitative data were expressions of gratitude and constructive feedback.

**Discussion:**

We report high patient satisfaction ratings in this first investigation of patient-centered outcomes in specialized interdisciplinary clinics for atypical parkinsonism. Future investigations focused on quality of life and care partner burden will provide additional insights for best care practices for people living with atypical parkinsonisms.

The progressive nature of symptoms and high level of disability experienced in atypical parkinsonian disorders renders no single clinical discipline equipped to adequately address the multifaceted needs of these patient populations. It is unknown whether interdisciplinary care in atypical parkinsonism is beneficial to patients and their care partners. Atypical parkinsonian disorders, including multiple system atrophy (MSA), progressive supranuclear palsy (PSP), and corticobasal syndrome (CBS), are life-limiting and rapidly progressive neurodegenerative diseases. These disorders typically present in the sixth to seventh decade of life, with an average survival of 8 years after symptom onset (Levin et al., 2016). People living with atypical parkinsonism experience a wide array of motor symptoms (postural instability and falls, ataxia, parkinsonism, and dystonia) and non-motor symptoms (autonomic failure, executive dysfunction, apraxia, sleep disturbance, bulbar symptoms, visual changes, pain, mood changes, and gastrointestinal and urinary problems) (Eschlböck et al., 2016; Levin et al., 2016). In the absence of disease-modifying treatments, clinical care remains largely focused on managing symptoms and optimizing quality of life (Bluett et al., 2021; Roach et al., 2020).

Over the course of disease progression, medical care for people with MSA, PSP, and CBS may include, but is not limited to, clinicians from subspecialized neurology (e.g., movement disorders, cognitive neurology), palliative care, urology, gastroenterology, neuropsychology, mental health, clinical social work, nursing, and rehabilitation therapy (Bluett et al., 2021; Eschlböck et al., 2016). Interdisciplinary care models have been described in the literature for progressive neurological diseases like Parkinson’s disease, Huntington’s Disease, and amyotrophic lateral sclerosis, yet the value of interdisciplinary care for patients with atypical parkinsonism remains understudied (Goldman et al., 2024; Khan et al., 2020; Lee et al., 2021; Moskowitz and Rao, 2017; Muzerengi et al., 2016; Rajan et al., 2020; Shahgholi et al., 2017; Talebi et al., 2023). There is limited but growing research describing the benefits of interdisciplinary care for atypical parkinsonian disorders (Clerici et al., 2017; Fabbri et al., 2024; Tilley et al., 2016).

One of the core concepts within interdisciplinary care models is patient centeredness, which is defined as the provision of care that is respectful of and responsive to individual preferences and ensures that the patient’s needs guide all clinical decisions (Epstein et al., 2010; McMillan et al., 2013). Understanding the patient and care partner’s experience and expectations has improved providers’ ability to facilitate better care (Bayen et al., 2022; Rapport et al., 2019; Wolf et al., 2008). Increasingly, patient experience questionnaires have been recognized as providing insight into the effectiveness of patient centeredness (O’Shea et al., 2024; Read et al., 2019; Rosqvist et al., 2019; Wensing and Elwyn, 2003). Studies have shown patients’ greater satisfaction with care leads to higher adherence with treatment plans and improved health outcomes (Jha et al., 2008; Manary et al., 2013).

The Atypical Parkinsonism Interdisciplinary Clinic at the University of North Carolina at Chapel Hill (UNC Chapel Hill) was established in 2017 with goals of streamlining coordination of care services, offering disease-specific education and tailoring treatment guidance for established patients with a possible or probable clinical diagnosis of MSA, PSP, and CBS. All patients who participate in the clinic are asked to complete an anonymous satisfaction survey after the interdisciplinary clinic visit. The survey was created as a quality improvement project within our center to provide feedback about the clinic to better serve our patients and improve the quality of care we provide. We define patients’ satisfaction with their care as a patient reported outcome measurement (Cella et al., 2015).

The objectives of this paper are to describe the Atypical Parkinsonism Interdisciplinary Clinic at UNC Chapel Hill, analyze the patient-reported outcome of satisfaction of care as a means of measuring the impact of our interdisciplinary model, and evaluate our internal clinical care practices. We hypothesized that the Atypical Parkinsonism Interdisciplinary Clinic patients would positively evaluate their experience of the clinic based on the categories of the patient satisfaction surveys: Services, Results, Clinicians, and Overall Experience. We further hypothesized that patients would have a better understanding of their disease overall after attending the specialized clinic.

## Methods

We conducted a retrospective, cross-sectional analysis of patients who attended the Atypical Parkinsonism Interdisciplinary Clinic at UNC Chapel Hill from 10/2017-3/2024, and of the anonymous patient satisfaction surveys that were distributed to all patients who were seen in the clinic in that date range. Approval for the research was granted through the University of North Carolina Institutional Review Board.

### Description of UNC Atypical Parkinsonism Interdisciplinary Clinic

The UNC Atypical Parkinsonism Interdisciplinary Clinic is located in Chapel Hill, North Carolina. Patients largely reside in the surrounding area as well as other parts of North Carolina and surrounding states. Most patients are referred to the interdisciplinary clinic after an initial evaluation by a movement disorders specialist at UNC Chapel Hill. The referrals are established patients with a possible or probable diagnoses of MSA, PSP, and CBS who:

• have limited rehabilitation experience
• new, worsening, or difficult-to-manage symptoms
• not receiving specialized care from clinicians familiar with MSA, PSP, and CBS.
• or those who seek more in-depth education about their disease.

**Figure 1.**
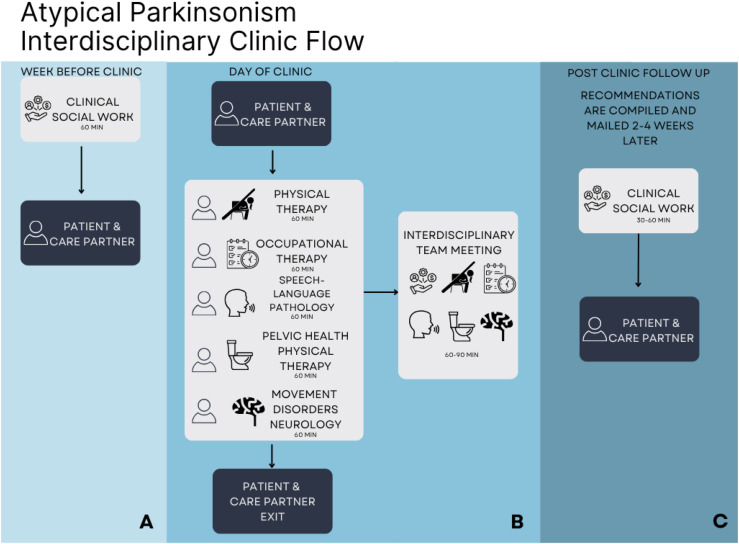
The timeline and flow of the UNC Atypical Parkinsonism Interdisciplinary clinic. (**A**) The clinical social worker contacts the patient and care partner to complete biopsychosocial assessment one week prior to the clinic. (**B**) The clinical assessment schedule for the patient and care partner (left side). After the patient and care partner exit all clinicians meet for an interdisciplinary team meeting (right side). (**C**) Post clinic follow up includes mailed summary and a phone call from the social worker to review and implement recommendations.

The interdisciplinary clinic flow is outlined in Figure 1. The clinical social worker serves as the coordinator of the clinic and helps to schedule patient appointments, connect patients with a financial counselor to review insurance coverage, and manages all necessary pre- and post-appointment paperwork. The clinic is held once a month. Patients who participate generally have strong motivation to attend, possess the necessary resources to travel to the center, and have the physical stamina to complete the five-hour clinical assessment. If patients have a care partner, they are encouraged to attend and remain engaged throughout the clinic visit. Prior to their appointment, patients complete several questionnaires aligned with the disciplines represented in the clinic (Table 1). Additional formal and informal assessments are conducted during the appointment as clinically indicated. All interdisciplinary clinic evaluations are individually billed to the patient’s insurance, with applicable co-pays and out-of-pocket costs. Patients without insurance may qualify for financial assistance through the university medical center.

**Table 1.** Evaluations conducted before, during, and after the clinic

Clinician	Evaluations conducted before the clinic	Evaluations conducted during the clinic	Post-clinic follow-up
Neurologist	Intake movement disorders questionnaire; UMSARS (Unified Multiple System Atrophy Rating Scale) part 1, COMPASS	Neurologic examination, movement disorders exam	Written feedback & recommendations
Clinical Social Worker	Biopsychosocial assessment, HADS (Hospital Anxiety and Depression Scale), Zarit Burden Interview, PSP QOL (Quality of Life) or MSA QOL as appropriate, Assessment of Goals for Clinic Participation, assessment of care partner’s needs and involvement in care	​	Written feedback & recommendations. Discussion with patient about interest in pursuing team’s recommendations and request to complete and return the satisfaction survey.
Physical Therapist	ABC Scale (Activities-specific Balance Confidence Scale)	M-PPT (Modified Physical Performance Test), 5x STS (Five-time Sit-to-St and test), 4 Square Step test, TUG (Timed Up and Go test), COG TUG (Cognitive Timed Up and Go test)	Written feedback & recommendations
Occupational Therapist	​	COPM (Canadian occupational performance measure) and the PPT (physical performance test) and Trail-Making Test, 9-hole peg test, Ocular Motor Exam, BiVaba	Written feedback & recommendations
Speech Language Pathologist	V-RQOL (Voice-Related Quality of Life Measure), EAT-10 (Eating Assessment Tool), Short IQCODE (Short Form of the Informant Questionnaire on Cognitive Decline in the Elderly),	clinical swallow evaluation, vocal quality and vocal volume assessments, MoCA (Montreal Cognitive Assessment)	Written feedback & recommendations
Pelvic Health Physical Therapist	Questionnaire reviewing Bowel and Bladder symptoms, sexual function concerns and history	pelvic alignment and hip screen assessment, and a pelvic floor assessment	Written feedback & recommendations

During the clinic visit, patients receive educational handouts and after visit summaries from each clinician. At the end of the visit, the interdisciplinary team convenes to discuss the patient’s primary goals, psychosocial context, diagnostic framework, and performance on assessments. After discussing each patient’s strengths, challenges, and needs the clinicians work together to develop a personalized care plan. The care plan, which typically includes recommendations for home exercise programs, referrals to additional evaluations, therapies or other specialists (e.g., modified barium swallow evaluation, neuro-ophthalmology, autonomic testing, palliative care), strategies for coping and care partner needs, adaptive equipment to maximize safety and independence with activities of daily living, and frequency of follow-up appointments with neurology and rehabilitation therapies, is written out in an easy-to-understand manner and mailed to the patient within two to four weeks of the clinic visit. The clinical social worker then follows up with the patient over the phone to answer questions, clarify and prioritize recommendations, and assist with referrals and resource coordination.

### Satisfaction Survey

**Figure 2.**
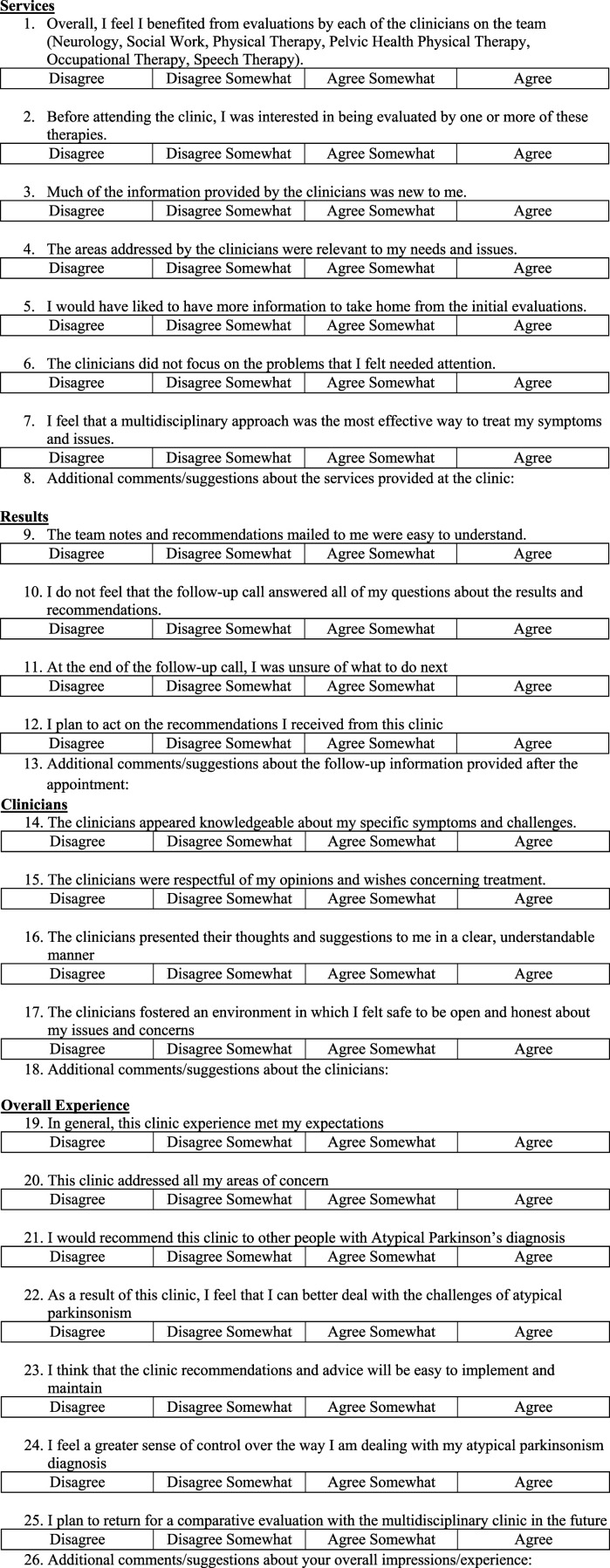
Patient satisfaction survey

To assess patient satisfaction with the clinic, a post-appointment survey is mailed to patients along with their care plan. The survey evaluates satisfaction with the interdisciplinary clinic in four categories: Services, Results, Clinicians, and Overall Experience and has a total of 26 questions (See Figure 2). Each section includes four to seven statements rated on a four – point Likert scale, along with an optional free text comment box for additional feedback. To encourage honest responses and reduce the chance for social desirability bias, the survey includes no identifying information, and a prepaid return envelope is provided to be mailed back to the clinic coordinator. All returned surveys were used in these analyses.

### Statistical Methods

Descriptive statistics (mean, percentages) were calculated for standard demographic data. Descriptive statistics were also analyzed for each questionnaire item and each category. Responses to each question on the satisfaction survey were converted to a linear scale ranging from –2 (disagree) to 2 (agree) for the purpose of quantitative analysis. We wanted to understand which categories are most likely to influence patient satisfaction. To investigate this, we performed linear regression analysis to assess predictive categories of the Overall Experience category score, which was felt to be reflective of the patients’ overall impression of the interdisciplinary clinic experience. The free-text responses were collected and analyzed by theme.

## Results

### Participants

The UNC Atypical Parkinsonism Interdisciplinary Clinic had 74 patients participate between October 2017 and March 2024. Patient characteristics can be found in Table 2. The cohort consisted of 48.6% males and 51.4% females, and ages ranged from 47-84 years (mean of 68.8 years). PSP represented the majority diagnosis of clinic patients at 51.4%, with MSA at 36.5%, CBS at 9.5%, Parkinsonism at 1.5%, and Parkinson’s disease at 1.5%.

**Table 2.** Patient Characteristics

​	n
Total patients	74
Men, n (%)	36 (48.6)
Women, n (%)	38 (51.4)
Age (years), mean (SD) [range]	68.7 (8.1) [47-84]
Ethnicity:	
Caucasian, n (%)	61 (82.4)
African American, n (%)	6 (8.1)
East Asian, n (%)	2 (2.7)
South Asian, n (%)	4 (5.4)
American Indian, n (%)	1 (1.4)
Diagnosis:	
MSA, n (%)	27 (36.5)
PSP, n (%)	38 (51.4)
CBS, n (%)	7 (9.5)
Parkinsonism, n (%)	1 (1.3)
PD, n (%)	1 (1.3)

### Patient Satisfaction

The patient satisfaction survey was completed by 39 of 74 total patients (52.7%). 6 surveys had 1-2 missing responses, and 1 survey had 4 missing responses; all available responses were included in analysis, missing values were counted as “0” which indicates a neutral score. Category scores are shown in Table 3, which indicate high levels of satisfaction with the care received by the interdisciplinary team. Responses indicated high levels of satisfaction across all categories, with 50% of survey question responses indicating high rated satisfaction.

**Table 3.** Results of the Patient Satisfaction Survey

​	Services	Results	Clinicians	Overall experience
Mean Total Score (possible score range)	10.21 (-14 - 14)	6.94 (-8 - 8)	7.46 (-8 – 8)	11.689 (-14 – 14)

In the Services category, patients reported high levels of satisfaction in 5 out of the 7 items. In two items there was less of a consensus. See Table 4.

**Table 4.** Services Category Items With More Variable Responses

5. I Would have liked to have more information to take home from the initial evaluations.			
**Disagree**	**Disagree Somewhat**	**Agree Somewhat**	**Agree**
9 (23.08%)	8 (20.51%)	9 (23.08%)	13 (33.33%)
6. The clinicians did not focus on the problems that I felt needed attention.			
**Disagree**	**Disagree Somewhat**	**Agree Somewhat**	**Agree**
32 (82.05%)	3 (7.69%)	2 (5.13%)	2 (5.13%)

In the Results category, participants mostly agreed with all four items indicating high satisfaction. Items 10 and 11 were phrased negatively, meaning participants’ disagreement with the question represented satisfaction with their care. 84.62% of participants disagreed with the statement “I do not feel that the follow-up call answered all of my questions about the results and recommendations.” 79.48% of participants disagreed with the statement “At the end of the follow-up call, I was unsure of what to do next.”

In the Clinicians category, participants agreed with all four items indicating high satisfaction. Only one patient disagreed with the statement “The clinicians appeared knowledgeable about my specific symptoms and challenges” in this section. The patients highly rated their experiences with the clinicians based on attitude, knowledge, and clarity of recommendations.

In the Overall Experience category, most patients agreed with all 7 items indicating high levels of satisfaction. 94.87% agreed that they would recommend the clinic to other patients with atypical parkinsonism. 94.87% of patients felt that the recommendations provided from the clinic will better help them deal with the challenges they face from their disease and 100% of patients agreed that the recommendations will be easy to implement and maintain in their lives.

**Figure 3.**
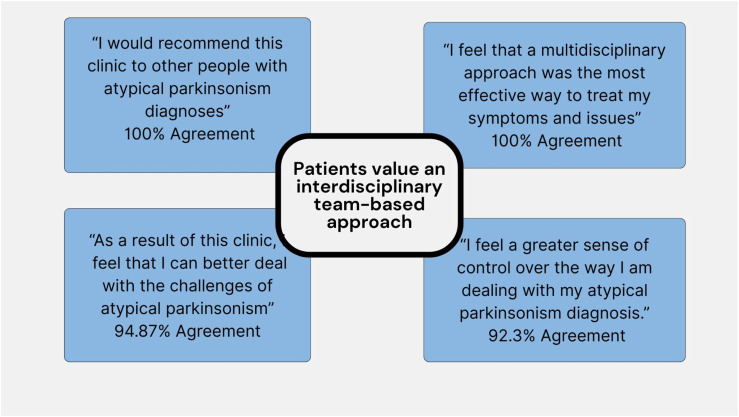
Illustrative survey questions regarding interdisciplinary care

Four questionnaire items were felt to be most reflective of the patients’ views on receiving interdisciplinary care (Figure 3). Based on responses to these questions, patients felt they benefited not only from attending our clinic but also because they believe an interdisciplinary team approach is beneficial in their care.

As a subanalysis, we were interested in understanding which components of the interdisciplinary clinic experience were most likely to result in a high level of overall satisfaction with our clinic. To do this, we performed multiple linear regression analyses to predict which of the category scores (Services, Results, or Clinicians) predicts the Overall Experience category score. The score in the Services category was shown to be the strongest predictor of the Overall Experience score ( β = 0.72, *p<*0.0001) and is the only section that was statistically significant. We realize this may be because Services has the most questions with the potential for the highest possible score. To best understand predictors of overall patient satisfaction scores, we chose to analyze responses to survey question 22, “As a result of this clinic, I feel that I can better deal with the challenges of atypical parkinsonism diagnosis.” This question was selected as it reflects the aims of our clinic to provide patients and families with disease-specific education, prepare for future care needs, and pursue therapies to manage symptoms and improve quality of life. No significant predictors were found.

### Thematic Comments

Of the surveys collected, 48.7% included responses to the free text survey items. Thematic analysis of these responses revealed six primary categories: positive experiences and expressions of gratitude, constructive feedback, disagreements with clinic notes, care partner concerns, uncertainty about the future, and billing issues.

The majority of responses (72%) reflected positive experiences and expressions of gratitude for the clinic. Common words and phrases included “knowledgeable,” “excellent,” “needed,” “helpful,” and “thank you.” Many respondents praised the team for their compassion and expertise.” Sample comments included: “They were nice, kind, approachable,” “All were outstanding!”, “This clinic was awesome! We came away much more informed and supported- needs addressed too! Thank all of you for what you do!”, and “It is good to have appropriate clinical information, and we are now better prepared to deal with issues.”

Constructive feedback appeared in 15% of responses. These comments suggested areas for improvement, including the need for more time with the pelvic health physical therapist, limited value for specific visits, and the importance of psychological support for those with PSP. Some comments noted delays in receiving the clinic notes, while others recommended logistical improvements like ensuring clinicians wear name tags or including more breaks between appointments.

Disagreement with the findings in the clinic notes was noted in 8.5% of responses. Patients expressed concern about perceived inaccuracies or performance on assessments, including the team’s comments about impulsivity, mood, handwriting, and work or driving status. Several patients felt that these remarks did not reflect their lived experience or functional abilities. A smaller proportion, at 4.25% of written responses, highlighted concerns regarding care partners, uncertainty about the future, and billing issues. One care partner expressed difficulty in gaining patient’s engagement, asking whether “getting ‘buy-in’ is a typical problem with PSP.” Another patient noted, “there is still a lot of unknown because of what it is,”, pointing to the ongoing challenges in understanding and managing the disease. Billing concerns were also raised, with at least one respondent stating, “billing was not accurate at all.”

Overall, the feedback offered valuable insights into both the strengths and areas for growth for the model and structure of the UNC Atypical Parkinsonism Interdisciplinary Clinic.

## Discussion

To our knowledge, this is the first study of patient-reported outcomes for interdisciplinary care for people with MSA, PSP, and CBS. Our findings demonstrate that the patients’ experience with the interdisciplinary clinic is overwhelmingly positive in each of the surveyed domains: Services, Results, Clinicians, and Overall Experience.

There are multiple factors positively influencing patients’ perceived satisfaction with their experience in this specialized interdisciplinary clinic. One of the most notable is the specialization and clinical expertise of the interdisciplinary team. In our study, the highly rated Clinicians section in the patient survey indicates that the high level of specialization of the clinicians contributed to overall satisfaction with the clinic. Many healthcare professionals lack familiarity with MSA, PSP, and CBS (Bluett et al., 2021; Roach et al., 2020) This can lead to significant delays in diagnosis as well as leave patients and families with feelings of uncertainty and discouragement as they strive to find clinicians with expertise in managing atypical parkinsonism symptoms and care (Wiblin et al., 2017; Respondek et al., 2023). Interdisciplinary care has also been shown to improve and enhance the overall education and specialization of the clinicians involved (Cohen et al., 2016).

In addition to clinician specialization, the structure of the interdisciplinary clinic allows patients and care partners to access multiple providers in one setting, reducing the burden on patient and care partner to advocate for their care needs, coordinate care between multiple providers and navigate fragmented care systems. Participation in a specialized interdisciplinary clinic allows for more streamlined and efficient management of a variety of symptomatic challenges (Lidstone, Bayley and Lang, 2020). In a study with late-stage Parkinson’s disease participants, who have similar needs to the atypical parkinsonism population, they compared satisfaction between patients who received care from an interdisciplinary team or not (Rosqvist et al., 2019). Those who received care from an interdisciplinary team reported higher satisfaction while only 36% of patients and care partners who did not receive specialized interdisciplinary care reported satisfaction with their care (Rosqvist et al., 2019). This is in stark contrast to the exceedingly high rates of satisfaction we encountered within our interdisciplinary clinic setting. Another study evaluating how people with late-stage Parkinson’s disease perceive their own care, discussed the importance of an interdisciplinary approach to care, though often left to the responsibility of the family to organize and coordinate (Read et al., 2019). That same study additionally found that families reported sensing a reluctance from clinicians to talk specifically about how to help their loved one and themselves plan for the future (Read et al., 2019). Our study demonstrated that patients seen in the interdisciplinary clinic felt satisfied with the care received from a streamlined and specialized team of clinicians as well as felt equipped to implement the recommendations, and to better cope with their disease progression.

There are a variety of disciplines that could be included in an interdisciplinary clinic to address the needs of atypical parkinsonism patients including but not limited to urology, ophthalmology, and palliative care. We do our best to address these gaps within our clinic design by utilizing the disciplines we have to make better referrals to additional specialists. For example, to address palliative care both neurology and social work address key elements such as advance care planning, symptom management, and a focus on quality of life. When a patient’s needs extend beyond the scope of these disciplines, we refer to specialized palliative care services for additional support. Our pelvic floor physical therapists provide insights and discuss opportunities for urology referrals and our occupational therapists can address and assess patient’s challenges with low vision and may suggest referrals for ophthalmology. While we are not able to include every discipline needed into this supplementary clinic we are able to provide this offering as an enhancement to address multiple challenges that would not be able to be covered in a patient’s standard neurological visit.

A person-centered approach has been a guiding principle of the UNC Atypical Parkinsonism Interdisciplinary Clinic. The assessments, team-based individualized recommendations, and follow-up conversations were designed to address patients’ physical, mental, and emotional needs. These measures also aim to integrate care partners into the discussion and care planning process. Understanding the subjective experiences of patients who are living with rare, rapidly progressing neurodegenerative diseases is necessary to optimize the quality of care provided. Previous studies have shown that patients who participate in a person-centered care model report positive experiences, better communication with their healthcare team, and increased education about their disease (Rapport et al., 2019; Wolf et al., 2008), which is also reflected in the results of our study. To best understand the full scope of patients’ experience we collected and analyzed both quantitative and qualitative data. Our findings, similar to previous work, found that negative comments often reflected lower quantitative scores; however, within our study those negative comments still produced overall high satisfaction ratings (Santuzzi et al., 2009).

Importantly, when we analyzed which aspects of the clinic visit contributed most strongly to overall satisfaction, the Services category was most predictive of the patient’s highly rated overall experience score. In this section, patients were asked about how they valued the care and recommendations they received from the clinicians during the clinic visit, and whether they felt these recommendations were relevant to their needs. This finding suggests that satisfaction is not just tied to clinical expertise but also to the delivery and tailoring of services to individual needs. We recognize that the services provided will vary from clinic to clinic and there is not a one-size-fits-all model of interdisciplinary care. There will be variability depending on the resources, discipline, and overall healthcare structure available to make this kind of patient-centered care possible (Lee et al., 2021), however, our findings reinforce the importance of aligning services with the priorities and goals of patients and families. Patient satisfaction and integrating the care experiences of patients into a patient’s care plan has become a key priority for health system reform in the United States (Geissler et al., 2013; Wensing and Elwyn, 2003). In comparable studies, patient dissatisfaction with care was expressed due to lengthy wait times for subspecialties like neurology or allied health professionals with knowledge about their disease or how to support them (Cyr et al., 2019; O’Shea et al., 2024; Read et al., 2019).

Our study is not without limitations. First, our response rate of 52.7% may introduce non-response bias, despite our efforts to reduce barriers to participation by providing prepaid return envelopes and an anonymous survey design. The response rate falls slightly below the typical average of 55–65% reported in mailed health surveys (Cook et al., 2000; Meyer et al., 2022). Because surveys were returned anonymously, we were unable to analyze response bias based on clinical or demographic variables. In future iterations of the survey, we plan to incorporate optional demographic questions to allow for more nuanced subgroup analysis without compromising anonymity. Second, while anonymity likely reduced social desirability bias, the inherent power dynamic between clinicians and patients may still have influenced how respondents answered (Bergen and Labonté, 2020). Third, as with all survey-based studies, patient satisfaction is a subjective construct, and, as such, there is ongoing debate in the literature regarding the validity and reliability of satisfaction measures (Al-Abri and Al-Balushi, 2014; Sitzia, 1999). Adaptation of a validated questionnaire to a clinic-specific satisfaction survey is a common practice, and many questions in our survey were designed to mirror validated questions (Cella et al., 2015; Larson, 1979). Nevertheless, the regular collection and thoughtful analysis of patient satisfaction data remains a cornerstone of many quality improvement initiatives and is a recommended strategy for aligning care with patient values and expectations (Al Abri and Al-Balushi, 2014; Bergen and Labonté, 2020; Cook et al., 2000; Geissler et al., 2013; Larsen et al., 1979; Meyer et al., 2022; Santuzzi et al., 2009; Sitzia, 1999; Larsen et al., 1979).

Finally, we also noted the larger range of scores for questions #5 and #6 in the Services category, and questions #10 and #11 in the Results section. These questions were negatively phrased, which may have been confusing for patients to answer as they changed the format of how the previous questions were stated in the survey. While this may have weakened the reliability of response to those questions, we felt it was important to mitigate acquiescence bias.

While our findings provide a strong case for the perceived value of interdisciplinary care for people with atypical parkinsonism, future studies should evaluate whether this model improves objective patient outcomes, such as quality of life, symptom burden, and healthcare utilization. Longitudinal follow-up and incorporation of care partner outcomes would further strengthen our understanding of the broader impact of this care model.

Our study contributes to the developing body of literature demonstrating the positive impact of interdisciplinary care with atypical parkinsonian disorders. While institution and clinic-specific barriers may exist that prevent exact replication of interdisciplinary clinic structures, our data indicate that specialized, team-based, and person-centered care are important components to providing highly satisfactory care for this complicated patient population. By outlining our center’s care model, we hope to facilitate a broader understanding of interdisciplinary care for MSA, PSP, and CBS ultimately resulting in patients and their families shaping their care, receiving more disease-specific education and feeling more equipped to navigate their disease journeys. We hope future research will replicate this interdisciplinary care model for people living with MSA, PSP, and CBS in ways that are unique to their center to improve quality of care.
